# Oscillatory dynamics of p38 activity with transcriptional and translational time delays

**DOI:** 10.1038/s41598-017-11149-5

**Published:** 2017-09-13

**Authors:** Yuan Zhang, Haihong Liu, Fang Yan, Jin Zhou

**Affiliations:** 1 Shanghai Institute of Applied Mathematics and Mechanics, Shanghai University, Shanghai, 200072 China; 2 Department of mathematics, Yunnan Normal University, Kunming, 650092 China

## Abstract

Recent experimental evidence reports that oscillations of p38 MAPK (p38) activity would efficiently induce pro-inflammatory gene expression, which might be deleterious to immune systems and may even cause cellular damage and apoptosis. It is widely accepted now that transcriptional and translational delays are ubiquitous in gene expression, which can typically result in oscillatory responses of gene regulations. Consequently, delay-driven sustained oscillations in p38 activity (p38*) could in principle be commonplace. Nevertheless, so far the studies of the impact of such delays on p38* have been lacking both experimentally and theoretically. Here, we use experimental data to develop a delayed mathematical model, with the aim of understanding how such delays affect oscillatory behaviour on p38*. We analyze the stability and oscillation of the model with and without explicit time delays. We show that a sufficiently input stimulation strength is prerequisite for generating p38* oscillations, and that an optimal rate of model parameters is also essential to these oscillations. Moreover, we find that the time delays required for transcription and translation in mitogen-activated protein kinase phosphatase-1 (MKP-1) gene expression can drive p38* to be oscillatory even when the concentration of p38* level is at a stable state. Furthermore, the length of these delays can determine the amplitude and period of the oscillations and can enormously extend the oscillatory ranges of model parameters. These results indicate that time delays in MKP-1 synthesis are required, albeit not sufficient, for p38* oscillations, which may lead to new insights related to p38 oscillations.

## Introduction

Mitogen-activated protein kinases (MAPKs), which play a key role in transducing various extracellular signals to the nucleus, are major signalling pathways of intracellular signalling circuitry^1–3^. At present, three major subgroups of MAPKs have been identified in higher eukaryotes, including extracellular signal-regulated kinase (ERK), c-Jun N-terminal kinase (JNK) and p38 MAPK (p38) families. Each MAPK pathway contains a three-tiered kinase cascade involving a MAPKK kinase (MAPKKK, MAP3K, MEKK or MKKK), a MAPK kinase (MAPKK, MAP2K, MEK or MKK) and the MAPK, that regulates various cellular activities such as proliferation, differentiation, apoptosis, survival, inflammation, and innate immunity^4–6^.

The p38 signalling pathway is famous for its importance in autoimmune and inflammatory diseases^7–10^, which consists of four isoforms (p38*α*, p38*β*, p38*γ* and p38*δ*) that are activated by numerous physical and chemical stresses, for example, hormones, ultraviolet, ischemia, cytokines (such as interleukin (IL)-1*β* and tumor necrosis factor (TNF)-*α*), osmotic shock and heat shock^4, 11^. In normal cells, the p38* is tightly controlled at a sustained low level^12^. In contrast, the p38* level is higher than a pre-stimulation baseline in response by stress conditions, which often leads to the inappropriate activation of pro-inflammatory gene that further results in damaging to the immune systems^13, 14^. As a result, suppression of p38* is a key mechanism to treat a variety of inflammations^15–18^. Oscillatory behavior, is an activation state of p38, which has been confirmed to play an important role in efficient expression of pro-inflammatory genes. Recently, using different temporal patterns of IL-1*β* stimulation to Hela cells, Tomida *et al*.^19^ reported that the oscillatory p38* induces pro-inflammatory genes more efficiently than non-oscillatory continuous p38*. More importantly, they discovered that the oscillation of p38* could potentially be deleterious to the cells and may even cause cellular damage and apoptosis. It is therefore important to identify the oscillatory mechanisms in p38* to control inflammatory diseases.

Typically, biochemical oscillations can be generated through delayed negative feedback. In particular, the delays involved in transcription and translation are critical^20–24^. It is known that transcriptional and translational processes are the basic steps of gene expression in cells, which are not just slow but also are compound multistage reactions involving the sequential assembly of long molecules^25^. As a result, both transcription and translation are usually related to long time delays^21, 26–28^. In general, there is an average transcriptional delay of around 10~20 min between the action of a transcription factor on the promoter of a gene and the appearance of the corresponding mature mRNA in the cytoplasm^29^. Similarly, a translational delay that takes around 1~3 min to synthesis of a typical protein from mRNA. Previously, oscillations driven by such delays have been extensively studied in several signaling pathways, such as in p53^30^, Her1 and Her7^24^. Nevertheless, up to today the effects of time delays on p38 pathway have not been reported. Specifically, a recent experiment has discovered that there exist transcriptional and translational time delays involving in the regulation of p38*, but the precise roles of such delays during the regulation process are still unclear^19^. Therefore, as an interesting and meaningful topic, it is tempting to explore the effects of these time delays to regulate the dynamical behaviour on p38*.

Motivated by the above considerations, we develop a mathematical model to explore the effects of time delays required for transcription and translation in MKP-1 on p38* oscillations. Using experimental data for human MKP-1, we firstly estimate the values of the total time delays for MKP-1 protein production between 9.4 and 30.7 min. To capture the underlying mechanisms of p38-MKP-1 interactions, we also investigate how different parameters can affect the dynamical behavior of the system. In particular, by introducing time delays into the signalling pathway, we find that the time delays during the regulatory process of MKP-1 gene expression are essential for sustained p38* oscillations. These results may advance our understanding of the oscillations of p38* and highlight the importance of considering time delays in regulation of the other MAPKs.

## Model formulation

**Figure 1:**
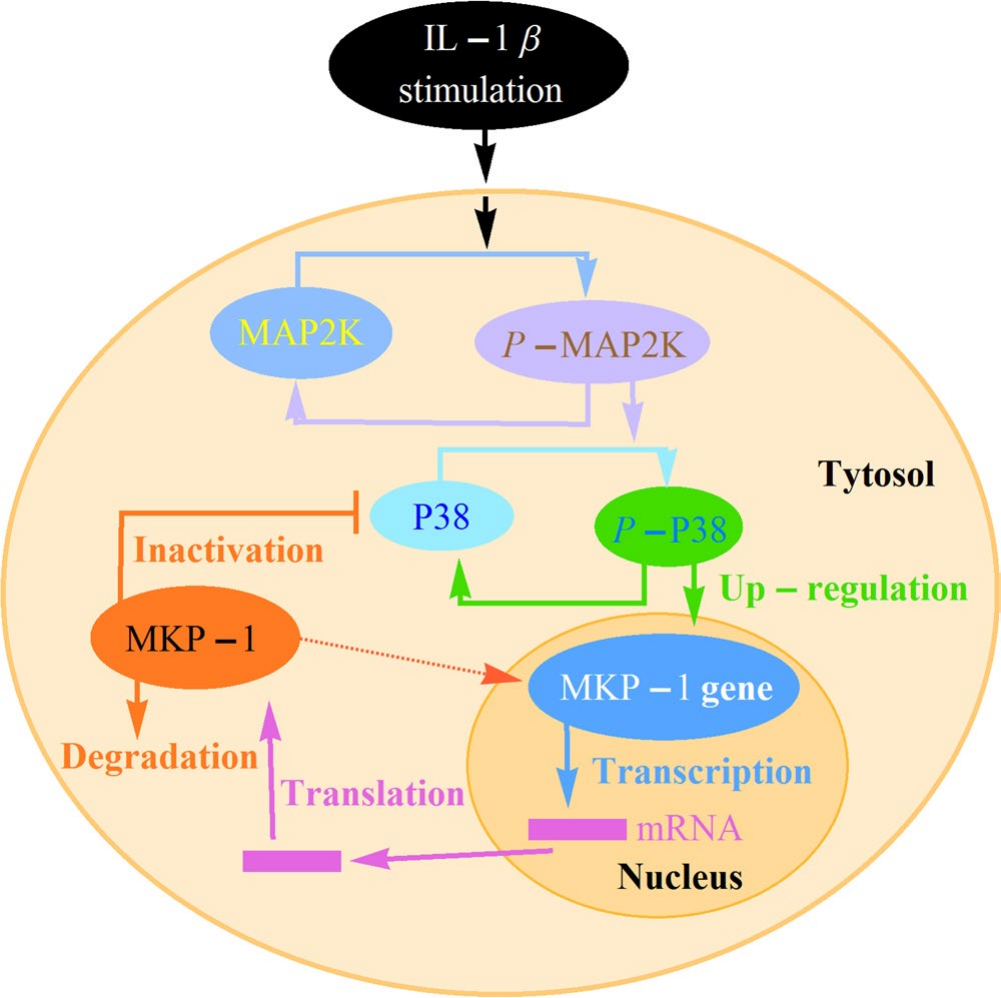
Experimentally determined network interactions among MAP2K, p38, MKP-1RNA and MKP-1 protein. Continuous IL-1*β* stimulation would activate MAPKK (MAP2K), leading to a subsequent activation of the p38. Subsequently, p38 phosphorylation can effectively induce the transcription of MKP-1 in the nucleus. MKP-1, is a major negative regulator of p38, which can inhibit the activity of p38 protein. Specifically, the synthesis of MKP-1 is a complex and time-consuming process that always exists inevitable time delays, from the initiation of transcription to the emergence of a complete functional MKP-1 protein molecule.

In Fig. 1, we provide a visual representation of the network of interactions among MAP2K, p38*, MKP-1RNA and MKP-1 protein, based on established biological facts and experimental data. When cells are exposed to continuous IL-1*β* stimulation, phosphorylation of MAPKK (MAP2K) occurs, which further leads to a subsequent activation of the p38 by phosphorylation at conserved threonine and tyrosine residues^19, 31^. Subsequently, p38 phosphorylation (p38*) can effectively induce the transcription of MKP-1 (also known as CL100, 3CH134, and Erp) in the nucleus. MKP-1, is a major negative regulator of p38, which can inhibit the activity of p38 to protect against cell damage from extracellular stress conditions^32–34^. However, it is important to note that the synthesis of MKP-1 is a complex and time-consuming process, which needs a certain time to complete the regulation of transcription and translation^19, 35^. As a result, there always exist inevitable time delays, from the initiation of transcription to the emergence of a complete functional MKP-1 protein molecule. Based on these properties, a detailed description of the model is given in the following.

Firstly, we modify the Tomida *et al*.’s model used to characterize the dynamics of the activation of MAP2K in response to stimulation signal:

1 $$\frac{dx(t)}{dt}={k}_{0}\times \frac{{s}^{{n}_{s}}}{{s}^{{n}_{s}}+{T}_{s}^{{n}_{s}}}\times \mathrm{(1}-x(t))-{k}_{1}\times x(t\mathrm{)}.$$

Here *x*(*t*) represents the concentration of the activity of the MAP2K at time *t*. The parameters *k*_0_ and *k*_1_ denote the rate of activation of MAP2K and the rate for MAP2K deactivation, respectively. Since the stimulatory input signal for inducing activation of MAP2K proposed in Tomida *et al*.^19^ is described by the Heaviside step function, the output of the model is not expected to offer a perfect recapitulation of the continuous stimulation response. In fact, experiment has suggested that stimulus/response data for MAP2K activation is as a Hill function^31^, $\frac{{s}^{{n}_{s}}}{{s}^{{n}_{s}}+{T}_{s}^{{n}_{s}}}$, in which *S* is the stimulatory input signal, *T*_*s*_ represents signal concentration for half-maximal MAP2K production, and *n*_*s*_ is a Hill coefficient of active MAP2K production by input stimulation signal.

The kinetics governing the concentration of p38*, *y*(*t*), are given by:

2 $$\frac{dy(t)}{dt}={k}_{2}\times x(t\mathrm{)(1}-y(t))-{k}_{3}\times z(t-{\tau }_{m})\frac{y(t)}{{k}_{4}+y(t)}.$$

Here the coefficient *k*_2_ denotes the rate of activation of p38, *k*_3_ specifies the maximum reaction rate of p38 inactivation by MKP-1, and *k*_4_ relates to an equivalent of the Michaelis-Menten constant for the reaction of MKP-1-mediated p38 inactivation. Parameter *τ*_*m*_ is a transcriptional time delay that includes the time required for transcript elongation, splicing, export and transport of messenger RNA in MKP-1 transcription. The quantity *z*(*t*) represents the protein concentration at time *t* of MKP-1. The second term reflects the degradation of p38* depending on MKP-1 level in a Michaelis-Menten form, in which, because the MKP-1 needs to take some time (transcriptional delay) to produce MKP-1 mRNA, the deactivation rate of p38 inactivated by MKP-1 depends on the concentration of MKP-1 at time *t* − *τ*_*m*_. Accordingly, the equation representing the kinetics of the MKP-1 protein expression level is given by

3 $$\frac{dz(t)}{dt}={k}_{7}\times w(t-{\tau }_{p})(1-z(t))-{k}_{8}\times z(t).$$

Here the coefficient *k*_7_ represents the rate constant for MKP-1 protein expression and *k*_8_ denotes the degradation rate for MKP-1 protein. Moreover, the discrete parameter *τ*_*p*_ is the translational time delay resulting from the translation of MKP-1 mRNA into MKP-1 protein. Here we assume that the rate of production of MKP-1 protein molecule depends on the concentration of MKP-1 mRNA at *t* − *τ*_*p*_ due to the time-consuming translational process^29^.

Finally, the equations to describe the kinetics of MKP-1 gene transcriptional level are given as follows:

4 $$\frac{dw(t)}{dt}={k}_{5}\times y(t)(1-w(t))-{k}_{6}\times w(t).$$

Here *w*(*t*) represents the concentration at time *t* of MKP-1 mRNA. *k*_5_ represents the rate constant for MKP-1 gene transcription, and *k*_6_ is the degradation rate for MKP-1 gene transcript. The first term in equation (4) describes the process that p38* promotes the transcription of MKP-1 gene, and the last term in equation (4) reflects the degradation of the MKP-1 mRNA.

In summary, building on Tomida *et al*. (2015)’s work, the equations used in the paper to model the dynamical relations of the network illustrated by Fig. 1 are as follows:

5 $$\begin{array}{rcl}\frac{dx(t)}{dt} & = & {k}_{0}\times \frac{{s}^{{n}_{s}}}{{s}^{{n}_{s}}+{T}_{s}^{{n}_{s}}}\times (1-x(t))-{k}_{1}\times x(t),\\ \frac{dy(t)}{dt} & = & {k}_{2}\times x(t)(1-y(t))-{k}_{3}\times z(t-{\tau }_{m})\frac{y(t)}{{k}_{4}+y(t)},\\ \frac{dw(t)}{dt} & = & {k}_{5}\times y(t)(1-w(t))-{k}_{6}\times w(t),\\ \frac{dz(t)}{dt} & = & {k}_{7}\times w(t-{\tau }_{p})(1-z(t))-{k}_{8}\times z(t).\end{array}$$

It is also noteworthy that, these time delays are incorporated based on the assumption that there is no degradation during the delayed processes^29, 30^. Moreover, due to the cyclic structure of system (5) and in the light of the approximations made in estimating time delays, we choose the *τ*_*m*_ and *τ*_*p*_ that affect the dynamical behaviors in the form of *τ* = *τ*_*m*_ + *τ*_*p*_. Interestingly, mathematical analysis also shows that the stability and the existence oscillation of p38* depend only on the total time delays. Therefore, in our study below, we will consider the effects of the total time delay *τ* = *τ*_*m*_ + *τ*_*p*_ on our model and do not differentiate whether *τ* appears in the transcriptional delay or in the translational delay.

Our model extends Tomida *et al*.’s work, with one key difference. That is, the effect of time delays on p38 pathway is ignored in the model proposed by Tomida *et al*., which has been taken as key consideration in our study. Previously, it was proposed that ignoring time delays in gene expression is questionable, except when the sum of them is much smaller than the other significant time scales characterizing the genetic system^36^. However, this constraint is generally not met in the transcription and translation of most proteins. For MKP-1 in human gene, which has a primary translation continuous open reading frame of length 1334 nt, with 3 introns, and codes for a protein of 367 amino acids^35^. Moreover, animal RNA polymerase II moves along DNA at an elongation rate of roughly 20 nucleotides per second^37, 38^; each successive intron takes between 0.4 and 7.5 min to splice out in mammalian cells^24, 39^; a further delay that nuclear mRNA emerges into the cytosol has been estimated at 4 min, and the mRNA is translated by ribosomes moving at roughly 6 nucleotides per second^39, 40^. Assuming the similar rates for human MKP-1, we estimate that the transcriptional delay is about 6.3~27.6 min and that the translational delay is about 3.1 min. Thus, we obtain that the estimated total delay time for MKP-1 protein production is between 9.4 and 30.7 min. Therefore, the model proposed by Tomida *et al*.^19^ can be reduced without the introduction of the time delays only when the remaining time scales are much longer than approximately 30 min. As a result, understanding such delays is essential for understanding how p38* is regulated except in extreme cases.

A phosphorylation cascade can have three different steady states, that is, stable low level state, stable high level state, and the sustained oscillation state^41^. To elucidate the biological meaning of the p38* level state, in the following, we suggest that if the level of p38* is at a low stable state, then the cell is at a controllable normal state^4, 15, 17, 19^; when the level of p38* is at a state of sustained oscillations, then the pro-inflammatory genes step into an efficient expression phase^19^. Moreover, because a high level of p38* can promote expression of many downstream genes to induce apoptosis^4, 15, 17^, we may suggest that if the p38* level is at a high stable state, then the cell undergoes irreversible apoptosis. Indeed, this conjecture seems to be consistent with the study that p38* induces neuronal apoptosis with high levels^17^. Additionally, a full list of model parameters and their default values is given in Table 1, and all the rate constants are used in all calculations, except where parameters are varied, or where noted otherwise. The bifurcation diagram is plotted by using the free softwares XPPAUT, and the other numerical simulations are performed by Mathematica 10.

**Table 1:** Parameters values for the mathematical model.

Rate constant	Interpretation	Value	Reference
*k*_0_	The rate constant for MAP2K activation	0.06 *min* ^−1^	19
*k*_1_	The rate constant for MAP2K deactivation	0.15 *min* ^−1^	19
*k*_2_	The MAP2K-induced synthesis rate for p38 activation	0.15 *min* ^−1^	19
*k*_3_	The maximum reaction rate of p38 inactivation by MKP-1	0.16 *min* ^−1^	19
*k*_4_	Michaelis constant of MKP-1-dependent p38 inactivation	0.0001	19
*k*_5_	The rate constant for MKP-1 gene transcription	0.055 *min* ^−1^	19
*k*_6_	The rate constant for degradation of MKP-1 gene transcript	0.05 *min* ^−1^	19
*k*_7_	The rate constant for MKP-1 protein expression	0.20 *min* ^−1^	19
*k*_8_	The rate constant for MKP-1 protein degradation	0.02 *min* ^−1^	19
*S*	The stimulatory input signal	10 *uM*	31
*T*_*s*_	Signal concentration for half-maximal p38 production	0.6 *uM*	31
*n*_*s*_	Hill coefficient of active MAP2K production by input stimulation signal	1.7	31
*τ*_*m*_	A transcriptional time delay	6.3~27.6 min	estimated
*τ*_*p*_	A translational time delay	3.1 min	estimated

## Results

### Dependence of dynamics on model parameters

To determine the significance of the time delays in the oscillations of p38*, an important step is to understand the dynamical behaviors of the non-delayed p38 system (in this case, the transcription and translation in system (5) are instantaneous processes, i.e., *τ* = 0). For this purpose, it is worthy to clarify the contributions of different model parameters to the p38* dynamics. Here, we mainly investigate the effects of four parameters, namely the stimulatory input signal strength, the MAP2K-induced synthesis rate of p38*, the strength of the negative feedback loop between p38 and MKP-1, and the degradation rate of MKP-1. Additionally, we provide a theoretical framework for the analysis, and some detailed results are given in Supporting Information Section 1 and Section 2.

**Figure 2:**
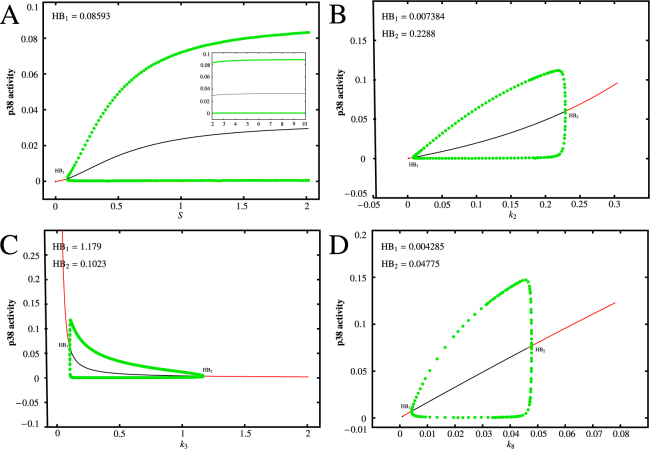
The effects of model parameters on the p38* dynamics. (**A**–**D**) The bifurcation diagram of p38* level versus *S* (**A**), that of p38* level versus *k*_2_ (**B**), that of p38* level versus *k*_3_ (**C**) and that of p38* level versus *k*_8_ (**D**). The red line represents stable state, the black line represents unstable state, and the green dots are the maxima and *min*ima of the stable limit cycles.

To broaden our understanding of the dynamics of the system, we firstly investigate the dynamics of the p38* in response to continuous stimulation. To show this, a bifurcation diagram of p38* level versus the stimulatory input signal strength, *S*, is given in Fig. 2A. At low basal stimulus, p38* remains predominantly in the inactive forms, and the corresponding steady state is stable. With the increasing input stimulation strength, p38* level also increases as a Hill function, well consistent with experimental stimulus/response data for MAPK activation^31^. Notably, there is a Hopf bifurcation at *S* = 0.08593, and beyond that p38* level can undergo periodical oscillations with nearly the same amplitude over a wide rang of *S*. This is consistent with the result that a threshold stimulus can switch the kinases into the active forms with sustained oscillations^41^. Moreover, if we set *S* = 0, namely there is no stimulation, then there are no sustained oscillations regardless of how changes of the model parameters are. These results indicate that a threshold level of input stimulation strength is prerequisite for generation of p38* oscillations.

Activated MAP2K can activate p38 through phosphorylation at conserved threonine and tyrosine residues, which can be quantified by the MAP2K-induced synthesis rate of p38*. To reveal the initiation mechanism for p38* oscillations, we plot the bifurcation diagram of p38* level versus *k*_2_, as shown in Fig. 2B. It is shown that p38* is so sensitive to its synthesis rate induced by MAP2K that a value 0.007384 is sufficient to induce p38* to be oscillatory. Moreover, the amplitudes of these oscillations increase with increasing *k*_2_ when the value of *k*_2_ is within a certain range (0.007384 < *k*_2_ < 0.2288). Furthermore, when *k*_2_ > 0.2288, it will fail to generate the oscillations, but the corresponding steady state is stable and high level. If high levels of p38* are of importance for an induction apoptosis, this finding implies that cells with abnormally high p38* basal activation rates should be associated with cellular apoptosis. Taken together, as the p38* synthesis rate goes from low values to higher values, the p38* level goes from a stable low level state into a series of sustained oscillations and then again to stable high level state. Therefore, a certain range of MAP2K-induced synthesis rates of p38* is required to generate the p38 oscillations.

Given the centrality of the p38-MKP-1 in treatment of inflammations, we further examine the role of the negative feedback loop in the generation of p38* oscillations, focusing on the influence of the coupling strength between p38 and MKP-1. Accordingly, Fig. 2C displays the bifurcation diagram of p38* level versus *k*_3_. For *k*_3_ < 0.1023, p38* level remains in a high steady state owing to weak inhibition of MKP-1, but drops rapidly with increasing *k*_3_. Moreover, there exist two Hopf bifurcation points at *HB*_1_ and *HB*_1_, respectively. As *k*_3_ increases gradually, a Hopf bifurcation labeled as *HB*_1_ appears at *k*_3_ = 0.1023, and beyond which p38* level undergoes periodical oscillations. When *k*_3_ is increased to the other Hopf bifurcation labeled as *HB*_2_ at *k*_3_ = 1.179, oscillations vanish. Importantly, one can see that the amplitudes of these oscillations from *HB*_1_ to *HB*_2_ drop with increasing *k*_3_. This result shows that the strength of the negative feedback loop between p38 and MKP-1 can not only inhibit p38* levels, but also can decrease the amplitude of the p38* oscillations. Interestingly, these results would be intriguing to examine whether enhancing the strength could destroy p38 oscillations in the response to stress conditions. Furthermore, when *k*_3_ > 1.179, p38* remains at low levels. Therefore, the strength of the p38-MKP-1 interactions significantly affects the dynamics of p38* and plays a pivotal role in the regulation.

Finally, it is worth to determine the role of the degradation rate of MKP-1, *k*_8_, in the regulation of p38*. Previously, Cloutier and Wang^42^ reviewed that the molecular mechanisms of mRNA or protein degradation are in general highly active in cancer cells, suggesting that the development of cancer treatments and prognostic markers could focus on the machinery of the molecular mechanisms of mRNA or protein degradation. This finding motivates the need to understand how MKP-1 degradation influences the dynamical behaviour of p38* to provide some clues for treatment of inflammation. Moreover, by taking *k*_8_ as a governing parameter, Tomida *et al*.^19^ demonstrated that the degradation rate can drive p38* to be oscillatory under continuous IL-1*β* stimulation; however, the underlying oscillatory mechanisms depending on the degradation rate are still not completely understood. Therefore, it is also important to clarify how the oscillations are regulated, and is necessary to further complement and improve the previous discussions. To do so, Fig. 2D shows the bifurcation diagram of p38* level versus *k*_8_. It can be seen that the p38* remains predominantly in a stable low level state when *k*_8_ is small enough (less than 0.004285 *min* ^−1^). As *k*_8_ increases, the p38* also increases gradually. In particular, when *k*_8_ passes through the critical value 0.004285, the p38* steady state loses its stability and a Hopf bifurcation occurs (at *HB*_1_), which leads to a series of sustained oscillations. Importantly, as previously mentioned, this demonstrates that pro-inflammatory genes step into an efficient expression phase in this scenario. Subsequently, when *k*_8_ is further increased to 0.04775, these oscillations disappear at the second Hopf bifurcation (at *HB*_2_). Furthermore, when *k*_8_ > 0.04775, p38* regains its stability but it turns into the stable high level state. In this situation, the level of p38* is much higher than the pre-stimulation baseline, which implies that the cell may undergo the irreversible process of apoptosis. These results well recapitulate the regulation mechanisms and functions of MKP-1 in inflammatory response^31, 43–45^, as well as the experimental and numerical results proposed by Tomida *et al*.^19^.

### Effects of time delays on oscillation of p38*

We next investigate the effects of the sum of the transcriptional and translational time delays on the oscillations of p38* with combination of theoretical approaches and dynamical simulations. To show this and to directly compare the results in Tomida *et al*.^19^, we begin with a particular example that the parameter *k*_8_ is satisfied *k*_8_ < 0.004285 *min* ^−1^ and *k*_8_ > 0.04775 *min* ^−1^ and the other parameters are fixed in Table 1 to confirm that sustained oscillations of p38* cannot be generated when there is no delay effects. Accordingly, in this case, *k*_8_ < 0.004285 *min* ^−1^ and *k*_8_ > 0.04775 *min* ^−1^ also correspond to two different biological scenarios, namely stable low and high level states of p38*, respectively.

To see whether such simple delays could generate oscillations of p38*, we firstly present certain oscillatory conditions for the model (5) using Hopf bifurcation technique (see the Supplementary Information Section 3). One can show analytically that if *k*_8_ satisfies certain conditions, then there exists a critical delay value *τ*_0_. By this *τ*_0_, it is demonstrated that the delay-driven oscillations of p38* can arose if the total time delays of the transcription and translation in MKP-1 expression surpass the critical value, unless p38* only exhibits stable state.

**Figure 3:**
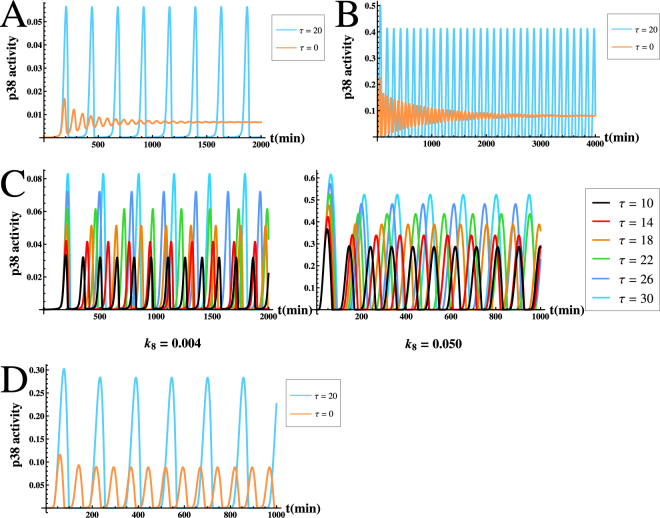
The effects of time delay *τ* on the p38* oscillations. (**A**) Time course of p38* with *k*_8_ = 0.004 *min* ^−1^ and *τ* = 20 *min*. (**B**) Time course of p38* with *k*_8_ = 0.050 *min* ^−1^ and *τ* = 20 *min*. (**C**) Impact of the delays on the amplitudes and periods of p38* oscillations. Both the amplitudes and the periods of the p38* oscillations increase with the increasing of *τ* for *k*_8_ = 0.004 and *k*_8_ = 0.050. (**D**) Impact of the delays on the oscillatory state of p38*. Under the regulation of transcriptional and translational time delays in MKP-1 protein production, the oscillation amplitudes and periods are larger then without delays. As an example, parameters *k*8 and *τ* are taken as 0.02 *min* ^−1^ and 20 *min*, respectively.

We now consider the first scenario that p38* is at a stable low level state. For this analysis we assume that *k*_8_ = 0.004 *min* ^−1^, which is the same rate as in Tomida *et al*.^19^. As previously shown, without the effects of transcriptional and translational time delays, the p38* lingers on a low stable level state as depicted in Fig. 2D. In contrast, under the effects of such delays, we can obtain that the critical value *τ*_0_ is 0.82 min. Clearly, it is significantly less than the estimated total delay time (9.4~30.7 min). As a result, the delay time (9.4~30.7 min) in protein production of MKP-1 is sufficient to drive the p38* to be oscillatory. As an example, Fig. 3A shows the time course of p38* with *τ* = 20 *min*. We can find that the periodic oscillations of p38* arose. Notably, this finding is consistent with the experimental observation that low level of p38* can display oscillatory behaviors and can also efficiently induce expression of pro-inflammatory genes^19^. Next, we turn to investigate the second scenario, in which the concentration of p38* is at a stable high level state. In this scenario, we take the degradation rate of MKP-1 as *k*_8_ = 0.050 *min* ^−1^. As we have seen (Fig. 2D), in the absence of delays that p38* has stepped into its high level stable state. Similar to the first scenario, we can obtain the critical value *τ*_0_ as 0.35 min that is also less than the estimated total delay time. Hence, we also find that the p38* exhibits sustained oscillations driven by such delays, as seen from Fig. 3B. Since the time delays can driven the high level stable state (corresponding to apoptosis) to be oscillatory (corresponding to an efficient pro-inflammatory genes expression state), this result may suggest that such delays could provide a safety mechanism to avoid cells to prematurely cause damage and apoptosis. Taken together, the time delays during the process of MKP-1 protein production can result in oscillations of p38* even when the concentration of p38* is at a stable low level state or at a stable high level state.

For thorough study the effectiveness of such delays on oscillatory p38*, we next consider the effects of the parameter *τ* variations on the amplitudes and periods of the oscillations by numerical simulations. As a example, Fig. 3C shows the time courses of p38* with different parameter delay *τ* for *k*_8_ = 0.004 *min* ^−1^ and *k*_8_ = 0.050 *min* ^−1^, respectively. It can be seen that both the amplitudes and the periods increase with the increasing of *τ*, particularly for *k*_8_ = 0.004 *min* ^−1^. Moreover, we also compare the time courses of p38 oscillatory state with or without the time delays as shown in Fig. 3D, in which *k*_8_ and *τ* are taken as 0.02*min* ^−1^ and 20 *min*, respectively. One can see that the oscillation amplitude and period with the delay effect are larger then without delays. Thus, by these simulations, we may conclude that both the amplitudes and the periods of the p38 oscillations are critically dependent on the total of the transcriptional and translational delays, suggesting that such delays might be used to control the amplitudes and periods in p38* oscillations.

**Figure 4:**
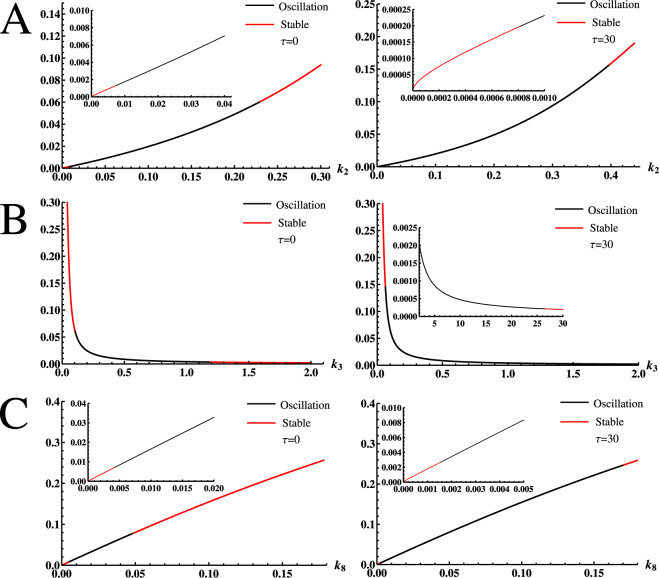
The effects of time delays on the oscillatory range of model parameters. Under the regulation of *τ* = 30, (**A**) the oscillatory range of *k*_2_ is from 0.007384~0.2288 *min* ^−1^ to 0.000812~0.39761 *min* ^−1^; (**B**) the oscillatory range of *k*_3_ is from 0.1023~1.179 *min* ^−1^ to 0.06233~26.6 *min* ^−1^; (**C**) the oscillatory range of *k*_8_ is from 0.004285~0.04775 *min* ^−1^ to 0.001548~0.16945 *min* ^−1^.

To more clearly show the effect of such delays in the p38 regulation, we next compare the oscillatory range of model parameters with and without delays. Under the regulation of time delays, we can calculate the maximal oscillatory range of *k*_8_ from the original range of 0.004285~0.04775 *min* ^−1^ into the range of 0.001548~0.16945 *min* ^−1^ as shown in Fig. 4C. Clearly, such delays can enlarge the oscillatory range of the parameter, particularly in the case of high degradation rate. Similarly, we can arrive at the following results: for *k*_2_, its oscillatory range is from 0.007384~0.2288 *min* ^−1^ to 0.000812~0.39761 *min* ^−1^ (Fig. 4C); for *k*_3_, its oscillatory range is from 0.1023~1.179 *min* ^−1^ to 0.06233~26.6 *min* ^−1^ (Fig. 4C), which increases about 24 times comparing to the oscillatory rang without time delays. Thus, we can conclude that the simple time delays in the MKP-1 protein production can enormously extend the oscillation ranges of the model parameters.

**Figure 5:**
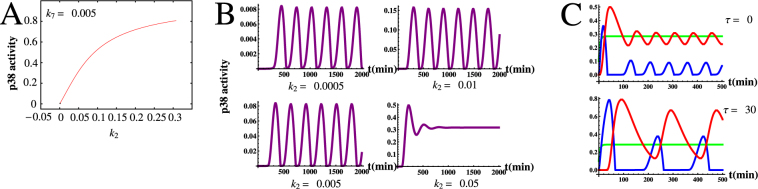
The effects of time delay *τ* on the p38* oscillations under low rates of MKP-1 translation. (**A**) The bifurcation diagram of p38* level versus *k*_2_ with *k*_7_ = 0.005. The same convention is used as in Fig. 2. (**B**) Time course of p38* with several fixed *k*_2_ values for *τ* = 30 *min*. (**C**) Time course of the levels of MAP2K (green), p38* (blue), and MKP-1 (red) with and without time delays.

As previously reported, MKP-1, which serves as an effective inhibitor of p38 protein, plays an important role in determining p38 oscillation. However, low rates of MKP-1 translation that will reduce the inhibition effect of MKP-1 for p38 because a lower translation rate results in a lower protein synthesis. Notably, the low rates of MKP-1 synthesis can lead to loss of p38* oscillations. Here, we also stress the significance of such delays in modulating p38* oscillations under low rates of MKP-1 translation. To show this and to compare with previous result, we plot a bifurcation diagram of p38* level versus *k*_2_ with *k*_7_ = 0.005 in Fig. 5A. Different from previous show, it can be seen that, for the low translation rate and without delay effects, p38* always remains in steady state over a wide rang of *k*_2_. However, interestingly, if we incorporate both the transcriptional and translational delays of MKP-1 into the regulation, sustained oscillations of p38* can be observed for some suitable values of *k*_2_ as shown in Fig. 5B. Therefore, under low rates of MKP-1 translation, a delay in MKP-1 synthesis is required for p38* oscillations.

Finally, it is worth comparing the dynamics among MAP2K, p38* and MKP-1 with and without time delays, in which model parameters are within the optimal intermediate ranges as given in Table 1. Accordingly, Fig. 5C respectively shows the time evolution of the three components with and without time delays. It is seen that, upon continuous stimulation, MAP2K expression quickly reaches a maximum level. Subsequently, both p38* and MKP-1 levels increase with respect to their basal levels whether there is time delay or not. Especially, the initial rise of p38* is due to the MAP2K promotion of p38 phosphorylation, and the initial rise of MKP-1 is because of the p38* induction of MKP-1 transcription. Next, due to the negative feedback loop between p38 and MKP-1, both the levels of p38* and MKP-1 start to oscillate in a sustained manner. In this particular example, MKP-1 peaks with a delay of ≈35 ± 5 min relative to p38*’s maximum, which is consistent with experimental data^19^. On the whole, under the sustained oscillation state, the dynamics among MAP2K, p38* and MKP-1 with and without time delays are similar in essence. The difference is that the regulation involving time delays makes the phase difference between p38* and MKP-1 is bigger, and results in a higher and wider p38* wave, which is more close to the experimental observations as shown in Fig. 2 proposed by Tomida *et al*.^19^.

## Discussion

We have developed a delayed model to study how transcriptional and translational delays in MKP-1 production affect dynamic behaviours on p38*. By analyzing the model both analytically and numerically, we firstly studied how the dynamics of p38* depend on the model parameters. We demonstrated that a threshold level of input stimulation strength may be prerequisite to initiate the p38* oscillations, and that a selected range of model parameters is also essential to the oscillations. In addition, by the delayed model, we showed that the direct autoinhibition with transcriptional and translational delays for MKP-1 by its own product can drive p38* to be oscillatory even when the concentration of p38* is at a stable low level state or at a stable high level state. Moreover, the length of these delays can determine the amplitude and period of the oscillations and can enormously extend the oscillation ranges of the model parameters. Our study complements the Tomida *et al*.’s^19^ work, indicating that time delays can have significant impact both on dynamical behavior and on parameter prediction of p38*.

As mentioned in the Introduction section, large oscillations of the p38* may potentially cause cellular damage and apoptosis. Our finding suggested that time delays in MKP-1 expression can determine the stability and the oscillations of p38*. Particularly, a larger time delay that would lead a larger oscillation. As a result, to ensure that delay-driven oscillation of p38* does not cause cellular damage or apoptosis, we need to reduce the total time delays to dampen the oscillations. An issue then arises concerning how the value of the total time delays is modulated. As far as we know, one effective way to shorten the total delay times is to delete the gene introns due to transcription and splicing of intron sequences increase the time necessary for mRNA production^26, 46^. This effective way is supported by experiments in recent studies^46, 47^. For instance, Takashima *et al*.^47^ experimentally tested that the Hes7 introns lead to an ~19-min delay in gene expression, demonstrating that without the intronic delay Hes7 oscillations would be abolished. Adopting the same approach to delete all of the three introns in MKP-1, we can reduce drastically the total time delays by 1.2~22.5 min based on the estimated time that it takes 0.4~7.5 min to splice out each intron^24, 39^. Thus, the number of the introns can be used to modulate the time delays required for MKP-1 gene expression.

Additionally, from the Results section, we have seen that the delay-driven oscillations of p38* is easy to produce. However, so far, experimental evidence of the oscillations has not been reported. In fact, the detection of the oscillations is difficult, requiring measurements with high temporal and spatial resolution^29^. Previously, it was proposed that such oscillations are not immediately obvious intuitively, but mathematics allows us to predict them^20^. Moreover, it is widely accepted now that only the stable limit cycle corresponding to oscillatory expression can be experimentally observed^21^. These motivate us to theoretically predict the feasibility and practical significance of our regulatory model. To do so, we given an explicit algorithm to determine the direction and stability of Hopf bifurcation for system (5) by applying the normal form theory and the center manifold reduction for functional differential equations developed by Hassard *et al*.^48^, as follows

6 $$\begin{array}{rcl}{c}_{1}\mathrm{(0)} & = & \frac{i}{2{\omega }_{0}{\tau }_{0}}({g}_{11}{g}_{20}-2{|{g}_{11}|}^{2}-\frac{{|{g}_{02}|}^{2}}{3})+\frac{{g}_{21}}{2},\\ {\mu }_{2} & = & -\frac{Re({c}_{1}\mathrm{(0))}}{Re(\lambda ^{\prime} ({\tau }_{0}))},\\ {\beta }_{2} & = & 2Re({c}_{1}\mathrm{(0)),}\\ {T}_{2} & = & -\frac{Im({c}_{1}\mathrm{(0))}+{\mu }_{2}Im(\lambda ^{\prime} ({\tau }_{0}))}{{\omega }_{0}{\tau }_{0}},\end{array}$$

which determine the properties of Hopf bifurcation. The detailed derivation and function of these indices are given in Supplementary Information Section 4. Here, as two particular examples, we considered *k*_8_ = 0.004 *min* ^−1^ and *k*_8_ = 0.050 *min* ^−1^, respectively. Accordingly, using the above three indices, we figured out that *μ*_2_ > 0, *T*_2_ > 0 and *β*_2_ < 0 for *k*_8_ = 0.004, 0.050 *min* ^−1^. Therefore, *μ*_2_ > 0 demonstrates that these two kinds of Hopf bifurcations are supercritical; *β*_2_ < 0 testifies that the bifurcating periodic solutions are stable; and *T*_2_ > 0 means that the periods of bifurcating periodic solutions are increasing. Thus, these results suggest that such delay-driven oscillation of p38* might be observed in real environment.

Finally, it is also noteworthy that for the sake of simplicity, the discrete delays *τ*_*m*_ and *τ*_*p*_ described by equations (5) are based on the assumption that the time taken from the initiation of transcription to the emergence of a complete functional MKP-1 protein molecule is exactly the same for every transcriptional/translational delay. Pragmatically, this assumption could be essential in understanding the whole process of gene regulation, because it is unrealistic to construct models that incorporate the numerous sequences of biochemical reactions that underlie the complexities of transcribing and translating a single gene^25^. Accordingly, such delays can effectively reduce the dimensionality of the complex process of gene expression and the difficulty of analytical and numerical techniques. More importantly, recent studies suggested that the discrete delays are more likely to generate oscillatory dynamics and are more easily verified by experiments^26, 47^. Thus, we believe this assumption proposed in our model is necessary and reasonable. In addition, we neglect the effect of the intrinsic noise in gene expression. In fact, such noise is also ubiquitous in regulation of gene expression^49^, which may be important under some conditions. For example, nonadiabatic noise from binding and unbinding of proteins to DNA has significant impact on predicting the region of the parameter space accommodating excitable behavior^50, 51^. Presumably, introducing intrinsic noise into our model will make the oscillatory amplitude and shape of p38* more random variation. Therefore, it would be intriguing to explore the integrated effects of delay and noise on p38 to provide more accurate theory for the treat of inflammatory diseases.

## Electronic supplementary material

Supplementary Information
