# Supplementary material for: Oscillatory dynamics of p38 activity with transcriptional and translational time delays
Source: Sci Rep. 2017 Sep 13;7:11495. doi: 10.1038/s41598-017-11149-5 (PMC5597677; doi:10.1038/s41598-017-11149-5)
Supplement: Supplementary file 1 — Supplementary Information [file 41598_2017_11149_MOESM1_ESM.pdf]

Supplementary Information

Oscillatory dynamics of p38 activity with transcriptional and translational time delays

Yuan Zhang[1]**, Haihong Liu**[2]**, Fang Yan**[2]**, and Jin Zhou**[1,*]

[1]Shanghai Institute of Applied Math and Mech, Shanghai University, Shanghai, 200072, China
[2]Department of mathematics, Yunnan Normal University, Kunming, 650092, China

ABSTRACT

The followings are supplemental results for "**Oscillatory dynamics of p38 activity with transcriptional and translational time delays**".

Delay model for p38 pathway

The basis of the model is as follows:

•Variables:
x(t), the activity of the MAP2Ks that activate p38.
y(t), the activity of the p38 MAPK.
w(t), the MKP-1 gene transcription level.
z(t), the MKP-1 protein expression level.
•Parameters:
k_0, the rate constant for MAP2K activation (0.06 min^{-1}).
k_1, the rate constant for MAP2K deactivation (0.15 min^{-1}).
k_2, the rate constant for p38 MAPK activation (0.15 min^{-1}).
k_3, the maximum reaction rate of p38 MAPK inactivation by MKP-1 (0.16 min^{-1}).
k_4, an equivalent of the Michaelis-Menten constant for the reaction of MKP-1-mediated p38 inactivation (0.0001).
k_5, the rate constant for MKP-1 gene transcription (0.055 min^{-1}).
k_6, the rate constant for degradation of MKP-1 gene transcript (0.05 min^{-1}).
k_7, the rate constant for MKP-1 protein expression (0.20 min^{-1}).
k_8, the rate constant for MKP-1 protein degradation (as a governing parameter).
S, the stimulatory input signal ($10uM$).
T_s, signal concentration for half-maximal p38 production ($0.6uM$)
n_s, Hill coefficient of active MAP2K production by input stimulation signal (1.7)
τ_m, the time required for transcript elongation, splicing, export and transport of messenger RNA (6.3 $\sim$ 27.6 min).
τ_p, the time required for translation of MKP-1 mRNA into MKP-1 protein (3.1 min).

Summary of the equations:

Here, for convenience, we collate the equations from the Model Formulation section of the main text. The "full model" is

governed by the following equations:

$$\begin{aligned}
\frac{dx(t)}{dt} &= k_0 \times \frac{s^{n_s}}{s^{n_s}+T_s^{n_s}} \times (1-x(t)) - k_1 \times x(t),\\
\frac{dy(t)}{dt} &= k_2 \times x(t)(1-y(t)) - k_3 \times z(t-\tau_m)\frac{y(t)}{k_4+y(t)},\\
\frac{dw(t)}{dt} &= k_5 \times y(t)(1-w(t)) - k_6 \times w(t),\\
\frac{dz(t)}{dt} &= k_7 \times w(t-\tau_p)(1-z(t)) - k_8 \times z(t).
\end{aligned} \tag{1}$$

1 Fixed point analysis

The system (1) has one unique positive fixed point denoted as (X^*, Y^*, W^*, Z^*), which satisfies the following equations

$$\begin{aligned}
&k_0 \times \tfrac{s^{n_s}}{s^{n_s}+T_s^{n_s}} \times (1-X^*) - k_1 \times X^* = 0,\\
&k_2 \times X^*(1-Y^*) - k_3 \times Z^* \frac{Y^*}{k4+Y^*} = 0,\\
&k_5 \times Y^*(1-W^*) - k_6 \times W^* = 0,\\
&k_7 \times W^*(1-Z^*) - k_8 \times Z^* = 0,
\end{aligned}$$

Therefore, we can obtain

$$\frac{k_0k_2S^{n_s}}{k_0T_s^{n_s}+k_0S^{n_s}+k_1S^{n_s}} - \frac{k_0k_2S^{n_s}Y^*}{k_0T_s^{n_s}+k_0S^{n_s}+k_1S^{n_s}} - \frac{k_3k_5k_7{Y^*}^2}{(k_4+Y^*)(k_6k_8+k_5k_7Y^*+k_5k_8Y^*)} = 0.$$

Let

$$H(Y) = \frac{k_0k_2S^{n_s}}{k_0T_s^{n_s}+k_0S^{n_s}+k_1S^{n_s}} - \frac{k_0k_2S^{n_s}Y}{k_0T_s^{n_s}+k_0S^{n_s}+k_1S^{n_s}} - \frac{k_3k_5k_7Y^2}{(k_4+Y)(k_6k_8+k_5k_7Y+k_5k_8Y)}.$$

It then follows that

$$H'(Y) = -\frac{k_0k_2S^{n_s}}{k_0T_s^{n_s}+k_0S^{n_s}+k_1S^{n_s}} - \frac{k_3k_5k_7Y(2k_4k_6k_8+k_4k_5k_7Y+k_4k_5k_8Y+k_6k_8Y)}{(k_4+Y)^2(k_6k_8+k_5k_7Y+k_5k_8Y)^2} < 0,$$

and $H(0) = \frac{k_0k_2S^{n_s}}{k_0T_s^{n_s}+k_0S^{n_s}+k_1S^{n_s}} > 0$ and $\lim_{P\to\infty} H(Y) < 0$, which implies that $H(Y)$ is monotonically decreasing in $(0, +\infty)$. Thus, we can conclude that $H(Y)$ has an unique positive root $Y^* \in (0, +\infty)$, and so system (1) has one unique positive fixed point (X^*, Y^*, W^*, Z^*).

2 Stability and bifurcations of p38 activity induced by model paramters

In this section, we give the detail mathematical analysis for the dynamics of non-delayed p38 system (1). By linearizing the system at the constant steady state (X^*, Y^*, W^*, Z^*), we can obtain the corresponding characteristic equation as follows

$$(\lambda + k_0\frac{s^{n_s}}{s^{n_s}+T_s^{n_s}} + k_1)(\lambda^3 + A_1\lambda^2 + A_2\lambda + A_3) = 0, \tag{2}$$

where

$$A_1 = k_6 + k_8 + k_7W^* + k_2X^* + k_5Y^* + \frac{k_3k_4Z^*}{(k_4+Y^*)^2} > 0,$$

$$\begin{aligned}
A_2 = {} & k_6k_8 + k_6k_7W^* + k_2k_6X^* + k_2k_8X^* + k_2k_7W^*X^* + k_5k_8Y^* + k_5k_7W^*Y^* + k_2k_5X^*Y^* + \frac{k_3k_4(k_6+k_8)Z^*}{(k_4+Y^*)^2}\\
& + \frac{k_3k_4(k_7W^*+k_5Y^*)Z^*}{(k_4+Y^*)^2} > 0,
\end{aligned}$$

$$\begin{aligned}
A_3 = {} & k_2k_6k_8X^* + k_2k_6k_7W^*X^* + k_2k_5k_8X^*Y^* + k_2k_5k_7W^*X^*Y^* + \frac{k_3k_4(k_8+k_7W^*)(k_6+k_5Y^*)Z^*}{(k_4+Y^*)^2} + k_3k_5k_7Y^*\\
& \times \frac{(1-W^*)(1-Z^*)}{k_4+Y^*} > 0.
\end{aligned}$$

As a example, we choose k_8 as the bifurcation parameter. Therefore, if the parameter k_8 satisfies the following condition

$$A_1A_2 - A_3 > 0, \tag{3}$$

then the fixed point (X^*, Y^*, W^*, Z^*) of system (1) is asymptotically stable.

Additionally, by Hopf bifurcation theorem, we obtain that when k_8 passes through the critical values $k_8^j (j \in N^+)$and satisfies $\left[\frac{d(Re(\lambda)}{dk_8}\right]|_{k_8=k_8^j} \neq 0$, then system (1) exhibits the Hopf bifurcation at (X^*, Y^*, W^*, Z^*), indicting that a family of periodic oscillations of p38 activity arose, in which k_8^j are the positive root of $A_1A_2 = A_3$.

Accordingly, using the above rate constants we obtain that there exist two Hopf bifurcation points at the degradation rate of MKP-1 values 0.004285 min^{-1} and 0.04775 min^{-1}, respectively.

3 Stability and Hopf bifurcations of p38 activity driven by time delays

Here, we take $\tau = \tau_m + \tau_p$ as a bifurcation parameter. By adopting Laplace transform, we now have the characteristic equation of linear model (1) at (X^*, Y^*, W^*, Z^*) as follows

$$(\lambda + k_0 \frac{s^{n_s}}{s^{n_s} + T_s^{n_s}} + k_1)(\lambda^3 + A_1\lambda^2 + A_2\lambda + a_1 + a_2 e^{-\lambda\tau}) = 0, \tag{4}$$

where A_1 and A_2 are given in Supplementary Information Section 2, and a_1 and a_2 are derived as follows:

$$a_1 = k_2k_6k_8X^* + k_2k_6k_7W^*X^* + k_2k_5k_8X^*Y^* + k_2k_5k_7W^*X^*Y^* + \frac{k_3k_4(k_8 + k_7W^*)(k_6 + k_5Y^*)Z^*}{(k_4 + Y^*)^2},$$

$$a_2 = \frac{k_3k_5k_7Y^*(1 - W^*)(1 - Z^*)}{k_4 + Y^*}.$$

To theoretically gain the sufficient conditions for the sustained oscillations, we assume that $i\omega$ be a root of equation (4), which implies that ω must satisfy the following equation

$$-i\omega^3 - A_1\omega^2 + iA_2\omega + a_1 + a_2(cos\omega\tau - isin\omega\tau) = 0,$$

which leads to

$$\omega^6 + (A_1^2 - 2A_2)\omega^4 + (A_2^2 - 2A_1a_1)\omega^2 + a_1^2 - a_2^2 = 0. \tag{5}$$

Moreover, it is easy to verify that

$$A_1^2 - 2A_2 > 0, \;\; A_2^2 - 2A_1a_1 > 0.$$

Therefore, if k_8 satisfies $a_1^2 < a_2^2$, then equation (5) has a unique positive root ω_0.

Define

$$\tau_0 = \frac{1}{\omega_0} arccos\left(\frac{A_1\omega_0^2 - a_1}{a_2}\right). \tag{6}$$

Thus, when $\tau = \tau_0$, equation (4) has a pair of purely imaginary roots $\pm i\omega_0$ and all of its other roots have negative real parts.

Furthermore, let $\lambda(\tau) = \nu(\tau) + \omega(\tau)i$ be a root of equation (4) near $\tau = \tau_0$ satisfying $\nu(\tau_0) = 0$ and $\omega(\tau_0) = \omega_0$, we have $\left[\frac{d(Re(\lambda)}{d\tau}\right]|_{\tau=\tau_0} > 0$. The detailed derivation is given as follows.

Substituting $\lambda(\tau)$ into the left hand side of (4) and taking derivative with respect to τ, we have

$$Re\left[\frac{d\lambda(\tau)}{d\tau}\right]^{-1}\Bigg|_{\tau=\tau_0} = \frac{3\omega_0^4 + 2(A_1^2 - 2A_2)\omega_0^2 + A_2^2 - 2A_1a_1}{B_1^2 + B_2^2},$$

where

$$B_1 = \omega_0^3 - A_2\omega_0,$$
$$B_2 = a_1 - A_1\omega_0^2.$$

Notice that $A_1^2 - 2A_2 > 0$ and $A_2^2 - 2A_1 a_1 > 0$, therefore

$$sign\left\{\left[\frac{d(Re(\lambda))}{d\tau}\right]\Big|_{\tau=\tau_0}\right\} = sign\left\{\left[\frac{d(Re(\lambda))}{d\tau}\right]^{-1}\Big|_{\tau=\tau_0}\right\} > 0.$$

Therefore, this result demonstrates that the roots of characteristic equation (4) cross the imaginary axis at $\lambda(\tau_0) = \pm i\omega_0$ from left to right. Clearly, system (1) undergoes a Hopf bifurcation at (X^*, Y^*, W^*, Z^*) when $\tau = \tau_0$.

As expected, combining the discussions above, we can obtain that when model parameters satisfy $a_1^2 < a_2^2$, then there exists a critical value τ_0. Moreover, if τ_0 is less than the total time delay of the transcriptional and translational delays in MKP-1 gene expression, it predicts that oscillations of p38 activity can arose, unless there only exhibits stable state. Obviously, the critical value τ_0 is essential to determine the stability and the oscillation of p38*.

4 Direction and stability of the Hopf bifurcation

To determine the direction of the Hopf bifurcations and the stability of bifurcated periodic solutions, we always assume that model (1) undergoes Hopf bifurcations at the positive fixed point (X^*, Y^*, W^*, Z^*) for $\tau = \tau_0$. Subsequently, let $\bar{x}(t) = x(t) - X^*$, $\bar{y}(t) = y(t) - Y^*$, $\bar{w}(t) = w(t) - W^*$, $\bar{z}(t) = z(t - \tau_m) - Z^*$ and denote $\bar{x}(t)$, $\bar{y}(t)$, $\bar{w}(t)$, $\bar{z}(t)$ by $x(t)$, $y(t)$, $w(t)$, $z(t)$. Moreover, rescaling the time by $t \to \frac{t}{\tau}$ to normalize the delay to 1, system (1) becomes

$$\begin{cases} \dfrac{dx(t)}{dt} = C_1 x, \\ \dfrac{dy(t)}{dt} = C_2 x + C_3 y + C_4 z - k_2 xy - G'(Y^*) k_3 yz - k_3 (z + Z^*) \sum_{i=2}^{\infty} \dfrac{1}{i!} G^{(i)}(Y^*) y^i, \\ \dfrac{dw(t)}{dt} = C_5 y + C_6 w(t) - k_5 wy, \\ \dfrac{dz(t)}{dt} = C_7 w(t-1) + C_8 z(t) - k_7 w(t-1) z, \end{cases} \tag{7}$$

where

$$C_1 = -(k_0 \frac{s^{n_s}}{s^{n_s} + T_s^{n_s}} + k_1), \;\; C_2 = k_2(1 - Y^*), \;\; C_3 = -k_2 X^* - G'(Y^*) k_3 Z^*, \;\; C_4 = -\frac{k_3 Y^*}{k_4 + Y^*},$$
$$C_5 = k_5(1 - W^*), \;\; C_6 = -k_6 - k_5 Y^*, \;\; C_7 = k_7(1 - Z^*), \;\; C_8 = -k_8 - k_7 W^*.$$

From system (1) or system (7), it can be seen that the activity of the MAP2Ks (x(t)) is independent of the activity of the p38 (y(t)), the MKP-1 gene transcription level (w(t)) and the MKP-1 protein expression level (z(t)). Therefore, to discuss the properties of Hopf bifurcation for system (1), we can only consider the following equations:

$$\begin{cases} \dfrac{dy(t)}{dt} = C_3 y + C_4 z - G'(Y^*) k_3 yz - k_3 (z + Z^*) \sum_{i=2}^{\infty} \dfrac{1}{i!} G^{(i)}(Y^*) y^i, \\ \dfrac{dw(t)}{dt} = C_5 y + C_6 w(t) - k_5 wy, \\ \dfrac{dz(t)}{dt} = C_7 w(t-1) + C_8 z(t) - k_7 w(t-1) z. \end{cases} \tag{8}$$

Denote $\tau = \gamma + \tau_0$, then $\gamma = 0$ is the Hopf bifurcation value of system (1) or system (8).

Let $U = (u_1(t), u_2(t), u_3(t))^T = (y(t), w(t), z(t))^T$ and define $\mathbf{C} = C([-1, 0], R^3)$. Then system (8) can be rewritten as

$$\dot{U} = L_\gamma(U_t) + f(\gamma, U_t), \tag{9}$$

where $L_\gamma : \mathbf{C} \to R^3$, $f : R \times \mathbf{C} \to R^3$ are respectively represented by

$$L_\gamma(\phi) = (\tau_0 + \gamma) \begin{pmatrix} C_3 & 0 & C_4 \\ C_5 & C_6 & 0 \\ 0 & 0 & C_8 \end{pmatrix} \begin{pmatrix} \phi_1(0) \\ \phi_2(0) \\ \phi_3(0) \end{pmatrix} + (\tau_0 + \gamma) \begin{pmatrix} 0 & 0 & 0 \\ 0 & 0 & 0 \\ 0 & C_7 & 0 \end{pmatrix} \begin{pmatrix} \phi_1(-1) \\ \phi_2(-1) \\ \phi_3(-1) \end{pmatrix} \tag{10}$$

and

$$f(\gamma,\phi)=(\tau_0+\gamma)\times\begin{pmatrix} -G'(Y^*)k_3\phi_1(0)\phi_3(0)-k_3(\phi_3(0)+Z^*)\sum_{i=2}^{\infty}\frac{1}{i!}G^{(i)}(Y^*)\phi_1(0)^i \\ -k_5\phi_1(0)\phi_2(0) \\ -k_7\phi_2(-1)\phi_3(0) \end{pmatrix}, \tag{11}$$

where $\phi=(\phi_1,\phi_2,\phi_3)^T\in\mathbf{C}$. By the Riesz representation theorem, there exists a 3×3 matrix function $\eta(\theta,\gamma),-1\le\theta\le 0$, whose elements are of bounded variation functions such that

$$L_\gamma\phi=\int_{-1}^{0}d\eta(\theta,\gamma)\phi(\theta)\ for\ \phi\in C([-1,0],R^3). \tag{12}$$

In fact, we can choose

$$\eta(\theta,\gamma)=(\tau_0+\gamma)\begin{pmatrix} C_3 & 0 & C_4 \\ C_5 & C_6 & 0 \\ 0 & 0 & C_8 \end{pmatrix}\delta(\theta)+(\tau_0+\gamma)\begin{pmatrix} 0 & 0 & 0 \\ 0 & 0 & 0 \\ 0 & C_7 & 0 \end{pmatrix}\delta(\theta+1) \tag{13}$$

where δ is a Dirac delta function. For $\phi\in C^1([-1,0],R^3)$, define

$$A(\gamma)\phi=\begin{cases} \frac{d\phi(\theta)}{d\theta}, & \theta\in[-1,0), \\ \int_{-1}^{0}\mathrm{d}\eta(\gamma,\theta)\phi(\theta), & \theta=0, \end{cases} \tag{14}$$

and

$$R(\gamma)\phi=\begin{cases} 0, & \theta\in[-1,0), \\ f(\gamma,\theta), & \theta=0. \end{cases} \tag{15}$$

Then system (9) is equivalent to

$$\dot{U}_t=A(\gamma)U_t+R(\gamma)U_t, \tag{16}$$

where $U_t(\theta)=U(t+\theta)$.

For $\psi\in C^1([0,1],(R^3)^*)$, define

$$A^*\psi(s)=\begin{cases} -\frac{d\psi(s)}{ds}, & s\in(0,1], \\ \int_{-1}^{0}\psi(-t)d\eta^T(t,0), & s=0, \end{cases} \tag{17}$$

and a bilinear inner product

$$\langle\psi(s),\phi(\theta)\rangle=\bar{\psi}(0)\phi(0)-\int_{-1}^{0}\int_{\xi=0}^{\theta}\bar{\psi}(\xi-\theta)d\eta(\theta)\phi(\xi)d\xi, \tag{18}$$

where $\eta(\theta)=\eta(\theta,0)$. Then $A(0)$ and $A^*(0)$ are adjoint operators. In addition, we know that $\pm i\omega_0\tau_0$ are eigenvalues of $A(0)$. Thus, they are also eigenvalues of $A^*(0)$. Let $q(\theta)$ be the eigenvector of $A(0)$ corresponding to $i\omega_0\tau_0$ and $q^*(s)$ is the eigenvector of $A^*(0)$ corresponding to $-i\omega_0\tau_0$.

Let $q(\theta)=(1,v_1,v_2)e^{i\omega_0\tau_0\theta}$ and $q^*(s)=G(1,v_1^*,v_2^*)\,e^{i\omega_0\tau_0 s}$. From the above discussion, it is easy to know that $A(0)q(0)=i\omega_0\tau_0 q(0)$ and $A^*(0)q^*(0)=-i\omega_0\tau_0 q^*(0)$. From Eq. (12), the definition of $\eta(\theta)$ and operator A, we have

$$\begin{pmatrix} C_3-\omega_0 i & 0 & C_4 \\ C_5 & C_6-\omega_0 i & 0 \\ 0 & C_7e^{-i\omega_0\tau_0} & C_8-\omega_0 i \end{pmatrix}\begin{pmatrix} 1 \\ v_1 \\ v_2 \end{pmatrix}=0, \tag{19}$$

and

$$\begin{pmatrix} C_3+\omega_0 i & C_5 & 0 \\ 0 & C_6+\omega_0 i & C_7e^{i\omega_0\tau_0} \\ C_4 & 0 & C_8+\omega_0 i \end{pmatrix}\begin{pmatrix} 1 \\ v_1^* \\ v_2^* \end{pmatrix}=0. \tag{20}$$

Thus, we can obtain

$$v_1=-\frac{C_5}{C_6-i\omega_0},\ \ v_2=-\frac{C_5C_7e^{-i\tau_0\omega_0}}{(iC_6+\omega_0)(iC_8+\omega_0)},$$

$$v_1^*=-\frac{C_4C_7e^{i\tau_0\omega_0}}{(-iC_6+\omega_0)(-iC_8+\omega_0)},\ \ v_2^*=-\frac{C_4}{C_8+i\omega_0}.$$

In order to ensure $\langle q^*(s),q(\theta)\rangle=1$, we need to determine the value of G. From (18), we have

$$\begin{aligned}\langle q^*(s),q(\theta)\rangle&=\overline{q}^*(0)q(0)-\int_{-1}^{0}\int_{\xi=0}^{\theta}\overline{q}^*(\xi-\theta)d\eta(\theta)q(\xi)d\xi\\&=\overline{q}^*(0)q(0)-\overline{q}^*(0)\int_{-1}^{0}\theta e^{i\omega_0\tau_0\theta}d\eta(\theta)q(0)\\&=\overline{q}^*(0)q(0)+\overline{q}^*(0)\tau_0\begin{pmatrix}0&0&0\\0&0&0\\0&C_7&0\end{pmatrix}e^{-i\omega_0\tau_0}q(0)\\&=\overline{G}\left[(1+v_1\overline{v}_1^*+v_2\overline{v}_2^*)+C_7v_1\overline{v}_2^*\tau_0e^{-i\omega_0\tau_0}\right]\end{aligned}$$

Thus we can choose G as

$$\overline{G}=\frac{1}{(1+v_1\overline{v}_1^*+v_2\overline{v}_2^*)+C_7v_1\overline{v}_2^*\tau_0e^{-i\omega_0\tau_0}},\tag{21}$$

where $\overline{G}$ is the conjugate imaginary of G.

Next we will compute the coordinate to describe the center manifold C_0 at $\gamma=0$. By using the same notions as those in Hassard et al.[1], let U_t be the solution of (16) when $\gamma=0$. Define

$$\begin{aligned}&z(t)=\langle q^*,x_t\rangle,\\&W(t,\theta)=U_t(\theta)-2Re\{z(t)q(\theta)\}.\end{aligned}\tag{22}$$

On the center manifold C_0, we have

$$W(t,\theta)=W(z(t),\overline{z}(t),\theta),$$

where

$$W(z,\overline{z},\theta)=W_{20}(\theta)\tfrac{z^2}{2}+W_{11}(\theta)z\overline{z}+W_{02}(\theta)\tfrac{\overline{z}^2}{2}+\cdots,\tag{23}$$

z and $\overline{z}$ are local coordinates for center manifold C_0 in the direction of q^* and $\overline{q}^*$. Note that W is real if x_t is real. We consider only real solution. For solution $U_t\in C_0$ of (16), since $\gamma=0$ and (22), we have

$$\begin{aligned}\dot{z}(t)&=\langle q^*,\dot{U}_t\rangle=\langle q^*,A(0)U_t+R(0)U_t\rangle\\&=\langle A^*(0)q^*,U_t\rangle+\langle q^*,f(0,U_t)\rangle\\&=i\omega_0\tau_0z+\overline{q}^*(0)f(0,W(z,\overline{z},\theta))+2Re\{z(t)q(\theta)\}\\&=i\omega_0\tau_jz+\overline{q}^*(0)f_0,\end{aligned}\tag{24}$$

that is,

$$\dot{z}(t)=i\omega_0\tau_jz(t)+g(z,\overline{z}),\tag{25}$$

where

$$g(z,\overline{z})=g_{20}\frac{z^2}{2}+g_{11}z\overline{z}+g_{02}\frac{\overline{z}^2}{2}+g_{21}\frac{z^2\overline{z}}{2}+\ldots.\tag{26}$$

Then it follows from (22) and (23) that

$$\begin{aligned}U_t&=W(t,\theta)+2Re\{z(t)q(\theta)\}=W_{20}(\theta)\tfrac{z^2}{2}+W_{11}(\theta)z\overline{z}+W_{02}(\theta)\tfrac{\overline{z}^2}{2}+zq+\overline{z}\overline{q}+\ldots.\\&=W_{20}(\theta)\tfrac{z^2}{2}+W_{11}(\theta)z\overline{z}+W_{02}(\theta)\tfrac{\overline{z}^2}{2}+(1,v_1,v_2)e^{i\omega_0\tau_0\theta}z+(1,\overline{v}_1,\overline{v}_2)e^{-i\omega_0\tau_0\theta}\overline{z}+\ldots.\end{aligned}\tag{27}$$

Substituting (11) and (27) into (26), we have

$$g(z,\bar{z}) = \overline{q}^*(0)f_0(z,\bar{z}) = \overline{q}^*(0)f(0,U_t)$$
$$= \overline{G}\tau_0(1,\overline{v}_1^*,\overline{v}_2^*)\begin{pmatrix} -G'(Y^*)k_3\phi_1(0)\phi_3(0)-k_3(\phi_3(0)+Z^*)\sum_{i=2}^{\infty}\frac{1}{i!}G^{(i)}(Y^*)\phi_1(0)^i \\ -k_5\phi_1(0)\phi_2(0) \\ -k_7\phi_2(-1)\phi_3(0) \end{pmatrix}.$$

Comparing the coefficients with (26), we obtain

$$g_{20} = 2\overline{G}\tau_0\Big(-\frac{1}{2}G''(Y^*)k_3Z^* - k_5v_1\overline{v_1^*} - G'(Y^*)k_3v_2 - e^{-i\tau_0\omega_0}k_7v_1v_2\overline{v_2^*}\Big),$$
$$g_{11} = \overline{G}\tau_0\Big(-G''(Y^*)k_3Z^* - k_5v_1\overline{v_1^*} - k_5\overline{v_1}\overline{v_1^*} - G'(Y^*)k_3v_2 - G'(Y^*)k_3\overline{v_2} - e^{i\tau_0\omega_0}k_7\overline{v_1}v_2\overline{v_2^*} - e^{-i\tau_0\omega_0}k_7v_1\overline{v_2}\overline{v_2^*}\Big),$$
$$g_{02} = 2\overline{G}\tau_0\Big(-\frac{1}{2}G''(Y^*)k_3Z^* - k_5\overline{v_1}\overline{v_1^*} - G'(Y^*)k_3\overline{v_2} - e^{i\tau_0\omega_0}k_7\overline{v_1}\overline{v_2}\overline{v_2^*}\Big),$$
$$g_{21} = 2\overline{G}\tau_0\Big(-\frac{1}{2}G'''(Y^*)k_3Z^* - G''(Y^*)k_3v_2 - \frac{1}{2}G''(Y^*)k_3\overline{v_2} - G''(Y^*)k_3Z^*W_{11}^1(0) - k_5v_1\overline{v_1^*}W_{11}^1(0)$$
$$-G'(Y^*)k_3v_2W_{11}^1(0) - k_5\overline{v_1^*}W_{11}^2(0) - k_7v_2\overline{v_2^*}W_{11}^2(-1) - G'(Y^*)k_3W_{11}^3(0) - e^{-i\tau_0\omega_0}k_7v_1\overline{v_2^*}W_{11}^3(0)$$
$$-\frac{1}{2}G''(Y^*)k_3Z^*W_{20}^1(0) - \frac{1}{2}k_5\overline{v_1}\overline{v_1^*}W_{20}^1(0) - \frac{1}{2}G'(Y^*)k_3\overline{v_2}W_{20}^1(0) - \frac{1}{2}k_5\overline{v_1^*}W_{20}^2(0) - \frac{1}{2}k_7\overline{v_2}\overline{v_2^*}W_{20}^2(-1)$$
$$-\frac{1}{2}G'(Y^*)k_3W_{20}^3(0) - \frac{1}{2}e^{i\tau_0\omega_0}k_7\overline{v_1}\overline{v_2^*}W_{20}^3(0)\Big)$$

Since there are $W_{20}(\theta)$ and $W_{11}(\theta)$ in g_{21}, we will need to compute them.

From (9) and (22), we have

$$\begin{aligned}\dot{W} &= \dot{U}_t - \dot{z}q - \dot{\bar{z}}\bar{q} \\ &= A(0)U_t + R(0)U_t - (i\omega_0\tau_0 z(t) + \bar{q}^*(0)f_0(z,\bar{z}))q(\theta) - [-(i\omega_0\tau_0\bar{z}(t) + q^*(0)\bar{f}_0(z,\bar{z}))\bar{q}(\theta)] \\ &= \begin{cases} AW - 2\Re\{\overline{q}^*(0)f_0q(\theta)\}, & \theta\in[-1,0) \\ AW - 2\Re\{\overline{q}^*(0)f_0q(\theta)\} + f_0, & \theta = 0 \end{cases} \\ &= AW + H(z,\bar{z},\theta),\end{aligned} \tag{28}$$

where

$$H(z,\bar{z},\theta) = H_{20}(\theta)\frac{z^2}{2} + H_{11}(\theta)z\bar{z} + H_{02}(\theta)\frac{\bar{z}^2}{2} + \ldots. \tag{29}$$

From (23), we have

$$\begin{aligned}\dot{W} &= \dot{W}_z\dot{z}(t) + \dot{W}_{\bar{z}}\dot{\bar{z}}(t) \\ &= (W_{20}(\theta)z + W_{11}(\theta)\bar{z} + \ldots)(i\omega_0\tau_0 z(t) + g(z,\bar{z})) + (W_{11}(\theta)z + W_{02}(\theta)\bar{z} + \ldots)(-i\omega_0\tau_0\bar{z}(t) + \bar{g}(z,\bar{z})).\end{aligned} \tag{30}$$

Substituting the corresponding series into (28), we obtain

$$\begin{aligned}\dot{W} &= A(0)(W_{20}(\theta)\tfrac{z^2}{2} + W_{11}(\theta)z\bar{z} + W_{02}(\theta)\tfrac{\bar{z}^2}{2} + \ldots) + H_{20}(\theta)\tfrac{z^2}{2} + H_{11}(\theta)z\bar{z} + H_{02}(\theta)\tfrac{\bar{z}^2}{2} + \ldots \\ &= (A(0)W_{20}(\theta) + H_{20}(\theta))\tfrac{z^2}{2} + (A(0)W_{11}(\theta) + H_{11}(\theta))z\bar{z} + (A(0)W_{02}(\theta) + H_{02}(\theta))\tfrac{\bar{z}^2}{2} + \ldots.\end{aligned} \tag{31}$$

Comparing the coefficients of z^2 and $z\bar{z}$ from (30) and (31), we get

$$\begin{aligned}(A(0) - 2i\omega_0\tau_0 I)W_{20}(\theta) &= -H_{20}(\theta), \\ A(0)W_{11}(\theta) &= -H_{11}(\theta).\end{aligned} \tag{32}$$

From (28), we know that for $\theta \in [-1,0)$,

$$\begin{aligned} H(z,\bar{z},\theta) &= -\overline{q}^*(0) f_0 q(\theta) - q^*(0)\overline{f}_0\overline{q}(\theta) \\ &= -g(z,\bar{z})q(\theta) - \overline{g}(z,\bar{z})\overline{q}(\theta) \\ &= -(g_{20}\tfrac{z^2}{2} + g_{11}z\bar{z} + g_{02}\tfrac{\bar{z}^2}{2} + \cdots)q(\theta) - (\overline{g}_{20}\tfrac{\bar{z}^2}{2} + \overline{g}_{11}z\bar{z} + \overline{g}_{02}\tfrac{z^2}{2} + \cdots)\overline{q}(\theta). \end{aligned} \tag{33}$$

Comparing the coefficients of z^2 and $z\bar{z}$ between (29) and (33), we obtain

$$H_{20}(\theta) = -g_{20}q(\theta) - \overline{g}_{02}\overline{q}(\theta) \tag{34}$$

and

$$H_{11}(\theta) = -g_{11}q(\theta) - \overline{g}_{11}\overline{q}(\theta). \tag{35}$$

From (32) and (34), we get

$$A(0)W_{20}(\theta) = 2i\omega_0\tau_0 W_{20}(\theta) - H_{20}(\theta).$$

From the definition of A(0), we have

$$\dot{W}_{20}(\theta) = 2i\omega_0\tau_0 W_{20}(\theta) + g_{20}q(\theta) + \overline{g}_{02}\overline{q}(\theta).$$

Note that $q(\theta) = q(0)e^{i\omega_0\tau_0\theta}$, hence we obtain

$$W_{20}(\theta) = \frac{ig_{20}}{\omega_0\tau_0}q(0)e^{i\omega_0\tau_0\theta} + \frac{i\overline{g}_{02}}{3\omega_0\tau_0}\overline{q}(0)e^{-i\omega_0\tau_0\theta} + E_1e^{2i\omega_0\tau_0\theta}, \tag{36}$$

where $E_1 = (E_1^{(1)}, E_1^{(2)}, E_1^{(3)})^T$ is a constant vector.

Similarly, from (32) and (35), we have

$$\dot{W}_{11}(\theta) = g_{11}q(\theta) + \overline{g}_{11}\overline{q}(\theta)$$

and

$$W_{11}(\theta) = -\frac{ig_{11}}{\omega_0\tau_0}q(0)e^{i\omega_0\tau_0\theta} + \frac{i\overline{g}_{11}}{\omega_0\tau_0}\overline{q}(0)e^{-i\omega_0\tau_0\theta} + E_2, \tag{37}$$

where $E_2 = (E_2^{(1)}, E_2^{(2)}, E_2^{(3)})^T$ is a constant vector. Now, we shall seek appropriate E_1 and E_2 in (36) and (37), respectively. It follows from the definition of A and (32) that

$$\int_{-1}^{0} d\eta(\theta)W_{20}(\theta) = 2i\omega_0\tau_0 W_{20}(\theta) - H_{20}(\theta) \tag{38}$$

and

$$\int_{-1}^{0} d\eta(\theta)W_{11}(\theta) = -H_{11}(0), \tag{39}$$

where $\eta(\theta) = \eta(0,\theta)$. From (28), we have

$$\begin{aligned} H_{20}(0) &= -g_{20}q(0) - \overline{g}_{02}\overline{q}(0)q(0) \\ &+2\tau_0 \begin{pmatrix} -\frac{1}{2}G''(Y^*)k_3Z^* - G'(Y^*)k_3v_2 \\ -k_5v_1 \\ -e^{-i\tau_0\omega_0}k_7v_1v_2 \end{pmatrix}, \end{aligned} \tag{40}$$

and

$$H_{11}(0) = -g_{11}q(0) - \overline{g}_{11}\overline{q}(0) + 2\tau_0 \begin{pmatrix} -G''(Y^*)k_3Z^* - G'(Y^*)k_3v_2 - G'(Y^*)k_3\overline{v_2} \\ -k_5v_1 - k_5\overline{v_1} \\ -e^{i\tau_0\omega_0}k_7\overline{v_1}v_2 - e^{-i\tau_0\omega_0}k_7v_1\overline{v_2} \end{pmatrix}. \tag{41}$$

Since $i\omega_0\tau_0$ is the eigenvalue of $A(0)$ and $q(0)$ is the corresponding eigenvector, then

$$\left(i\omega_0\tau_0 I - \int_{-1}^{0} e^{i\omega_0\tau_0\theta} d\eta(\theta)\right) q(0) = 0$$

and

$$\left(-i\omega_0\tau_0 I - \int_{-1}^{0} e^{-i\omega_0\tau_0\theta} d\eta(\theta)\right) \overline{q}(0) = 0.$$

Therefore, we have

$$\begin{pmatrix} 2i\omega_0 - C_3 & 0 & -C_4 \\ -C_5 & 2i\omega_0 - C_6 & 0 \\ 0 & -C_7 e^{-2i\omega_0\tau_0} & 2i\omega_0 - C_8 \end{pmatrix} \times E_1 = 2 \begin{pmatrix} -\frac{1}{2}G''(Y^*)k_3 Z^* - G'(Y^*)k_3 v_2 \\ -k_5 v_1 \\ -e^{-i\tau_0\omega_0} k_7 v_1 v_2 \end{pmatrix}, \tag{42}$$

and

$$\begin{pmatrix} -C_3 & 0 & -C_4 \\ -C_5 & -C_6 & 0 \\ 0 & -C_7 & -C_8 \end{pmatrix} \times E_2 = 2 \begin{pmatrix} -G''(Y^*)k_3 Z^* - G'(Y^*)k_3 v_2 - G'(Y^*)k_3 \overline{v_2} \\ -k_5 v_1 - k_5 \overline{v_1} \\ -e^{i\tau_0\omega_0} k_7 \overline{v_1} v_2 - e^{-i\tau_0\omega_0} k_7 v_1 \overline{v_2} \end{pmatrix}, \tag{43}$$

Therefore, we can determine $W_{20}(\theta)$ and $W_{11}(\theta)$ from (36) and (37). Furthermore, g_{21} can be expressed. Thus, we can compute the following values:

$$\begin{aligned} c_1(0) &= \frac{i}{2\omega_0\tau_0}\left(g_{11}g_{20} - 2|g_{11}|^2 - \frac{|g_{02}|^2}{3}\right) + \frac{g_{21}}{2}, \\ \mu_2 &= -\frac{Re(c_1(0))}{Re(\lambda'(\tau_0))}, \\ \beta_2 &= 2Re(c_1(0)), \\ T_2 &= -\frac{Im(c_1(0)) + \mu_2 Im(\lambda'(\tau_0))}{\omega_0\tau_0}. \end{aligned} \tag{44}$$

In (44), we have the following results:

(1) The direction of Hopf bifurcation is determined by μ_2: if $\mu_2 > 0$ ($resp.$ $\mu_2 < 0$), then the Hopf bifurcation is supercritical (resp. subcritical) and the bifurcating periodic solutions exist for $\tau > \tau_0$ ($resp.$ $\tau < \tau_0$);

(2) The stability of the bifurcating periodic solutions is determined by β_2: the bifurcating periodic solutions in the center manifold are stable (resp. unstable) if $\beta_2 < 0$ ($resp.$ $\beta_2 > 0$);

(3) The period of the bifurcating periodic solutions is determined by T_2: the period increases (resp. decreases) if $T_2 > 0$ ($resp.$ $T_2 < 0$).

To investigate the stability of oscillation, using the above three indices in (44), we obtain the following results: for $k_8 = 0.004$, we figure out $\mu_2(\tau = \tau_0) = 221704$, $T_2(\tau = \tau_0) = 16139.2$, $\beta_2(\tau = \tau_0) = -1193.45$; for $k_8 = 0.050$, we have $\mu_2(\tau = \tau_0) = 21.7125$, $T_2(\tau = \tau_0) = 1.45463$, $\beta_2(\tau = \tau_0) = -0.120763$. Therefore, $\mu_2 > 0$ demonstrates that these two kinds of Hopf bifurcations are supercritical; $\beta_2 < 0$ testifies that the bifurcating periodic solutions are stable; and $T_2 > 0$ means that the periods of bifurcating periodic solutions are increasing. These results predict that such delay-driven oscillation of p38 activity might be observed in real environment.

References

1. Hassard,B.D. *et al.* (1981) The Hopf bifurcation theorem. *Theory and applications of Hopf bifurcation*. Vol. 41, Cambridge University Press, Cambridge, pp. 14-71.
